# Supplementary material for: The genome sequence of the Downland Conch, Aethes tesserana (Denis & Schiffermüller) , 1775 (Lepidoptera: Tortricidae)
Source: Wellcome Open Res. 2026 May 11;11:273. [Version 1] doi: 10.12688/wellcomeopenres.26512.1 (PMC13329360; doi:10.12688/wellcomeopenres.26512.1)
Supplement: Peer review history

# Reviewer report: Reviewer response for version 1

**DOI:** 10.21956/wellcomeopenres.29197.r156054

**Published:** 2026-07-16

**Authors:** Annabel Whibley

**Affiliations:** 1 Bragato Research Institute, Lincoln, New Zealand

This Data Note reports a reference genome assembly of the Downland Conch, *Aethes tesserana*. I think it should be more clearly noted that the image in Figure 1 of the Data Note is not the sequenced individual (perhaps removing the link to Figure 1 in the methods would achieve this). The identity of the specimen was, however, verified by DNA barcoding.  This minor concern aside, the report follows the standard data combination (HiFi plus HiC), sample preparation, sequencing, assembly and evaluation pipeline. Reporting is comprehensive and methods are appropriate. The genome quality is very high with no metrics hinting at any underlying issues. The noting of scaffolded contigs that are of uncertain order/orientation is appreciated.

Are sufficient details of methods and materials provided to allow replication by others?

Yes

Is the rationale for creating the dataset(s) clearly described?

Yes

Are the datasets clearly presented in a useable and accessible format?

Yes

Are the protocols appropriate and is the work technically sound?

Yes

Reviewer Expertise:

Bioinformatics, Genomics

I confirm that I have read this submission and believe that I have an appropriate level of expertise to confirm that it is of an acceptable scientific standard.

# Reviewer report: Reviewer response for version 1

**DOI:** 10.21956/wellcomeopenres.29197.r158771

**Published:** 2026-07-02

**Authors:** Jaakko Pohjoismäki

**Affiliations:** 1 University of Eastern Finland, Joensuu, Finland

The Natural History Museum Genome Acquisition Laboratory and the Darwin Tree of Life Consortium present a chromosome-level reference genome assembly for the tortricid moth Aethes tesserana. This small, distinctive, and visually striking species is also of conservation interest, being endangered in parts of its European range, making this assembly a valuable addition to the growing collection of high-quality lepidopteran genomes.

 The sequencing, assembly, and curation follow the established Darwin Tree of Life pipeline and have resulted in a highly contiguous and high-quality genome assembly. The genome was generated from approximately 85× sequencing coverage, with one haplotype assembled and manually curated to chromosome level and the second assembled to scaffold level. The final primary assembly spans 486.1 Mb in only 69 scaffolds, achieving a scaffold N50 of 17.5 Mb. Assembly quality metrics are excellent, with an estimated QV of 65.6 for the primary haplotype (65.9 for the combined assembly), a combined k-mer completeness of 99.2%, and a BUSCO completeness of 98.5%.

 The manuscript is clearly written and the assembly metrics demonstrate that this is a valuable genomic resource. The background could be more extensive, as it currently provides a rather brief overview of the species. Expanding this section with a few sentences highlighting e.g. the potential applications of the genome assembly place this resource into a broader biological context and further increase the impact of the genome note.

Are sufficient details of methods and materials provided to allow replication by others?

Yes

Is the rationale for creating the dataset(s) clearly described?

Yes

Are the datasets clearly presented in a useable and accessible format?

Yes

Are the protocols appropriate and is the work technically sound?

Yes

Reviewer Expertise:

Molecular biology and genetics; species diversity; biodiversity genomics

I confirm that I have read this submission and believe that I have an appropriate level of expertise to confirm that it is of an acceptable scientific standard.

# Reviewer report: Reviewer response for version 1

**DOI:** 10.21956/wellcomeopenres.29197.r158767

**Published:** 2026-06-26

**Authors:** Jurate De Prins

**Affiliations:** 1 National Collections and Marine Infrastructure, Commonwealth Scientific and Industrial Research Organisation, Leefdaal, Vlaams-Brabant, Belgium

From a taxonomic point of view, I have two comments:

 1) The title of the article is not correct. I have a great pleasure of possessing the book "Ankündung eines systematischen Werkes von den Schmetterlingen der Wienergegend herausgegeben von einigen Lehrern am k. k. Theresianum.", published by Augustin Bernardi, Wien, written by two teachers who were too modest to put their names as authors of this book. Later, the authorship was established as Denis & Schiffermüller. Therefore, *Aethes tesserana* authorship ****should be cited as follows: ([Denis & Schiffermüller], 1775). If the authors do not want to use square brackets (additions of the ICZN Code allow this), then they may use the combination only with parentheses *Aethes tesserana* (Denis & Schiffermüller, 1775). In both cases, the presentation as it is in the title *Aethes tesserana* (Denis & Schiffermüller), 1775 is incorrect.

 2) *Aethes tesserana* is a very peculiar micromoth and can be identified from external characters. Many micromoths, also in Europe, can be identified only by internal characters of genitalia. It is highly advisable to include genitalia comparison for micro moth species ID into the protocol that deals with small moths, which in some cases might be invasive.

Are sufficient details of methods and materials provided to allow replication by others?

Yes

Is the rationale for creating the dataset(s) clearly described?

Yes

Are the datasets clearly presented in a useable and accessible format?

Yes

Are the protocols appropriate and is the work technically sound?

Yes

Reviewer Expertise:

Taxonomy, microlepidoptera, karyology

I confirm that I have read this submission and believe that I have an appropriate level of expertise to confirm that it is of an acceptable scientific standard, however I have significant reservations, as outlined above.

## References

[1] Denis, M. & Schiffermüller, I.1775. Ankündung eines systematischen Werkes von den Schmetterlingen der Wienergegend herausgegeben von einigen Lehrern am k. k. Theresianum. Augustin Bernardi, Wien, i, 1–323, pls 1–3.

# Reviewer report: Reviewer response for version 1

**DOI:** 10.21956/wellcomeopenres.29197.r155459

**Published:** 2026-05-29

**Authors:** Fedor Čiampor

**Affiliations:** 1 Department of Biodiversity & Ecology, Plant Science and Biodiversity Centre, Slovak Academy of Sciences, Bratislava, Slovakia

This data note presents a high-quality genome assembly for the moth Aethes tesserana produced within the Darwin Tree of Life project. The contribution is scientifically useful, the sequencing and assembly workflow is clearly described.

 The assembly quality appears strong, the inclusion of raw data accessions, software versions, and deposition information also supports reproducibility and reuse.

 Only minor improvements are needed. The manuscript could briefly explain the biological or comparative value of the species, i.e. how can genome data be used. It would also be useful to clarify the impact of the two noted regions with uncertain contig order/orientation on chromosomes 18 and 26. Finally, in the title there is “(Denis & Schiffermüller), 1775”, but it should be “(Denis & Schiffermüller, 1775)”Overall, this is a concise, well-documented and valuable genome resource suitable for indexing after minor clarification.

Are sufficient details of methods and materials provided to allow replication by others?

Yes

Is the rationale for creating the dataset(s) clearly described?

Yes

Are the datasets clearly presented in a useable and accessible format?

Yes

Are the protocols appropriate and is the work technically sound?

Yes

Reviewer Expertise:

Arthropod DNA (meta)barcoding

I confirm that I have read this submission and believe that I have an appropriate level of expertise to confirm that it is of an acceptable scientific standard.
